# cGAS-STING/HMGB1-mediated senescence induced by LRRK2 accelerates cartilage degeneration in osteoarthritis

**DOI:** 10.1038/s41419-026-08651-y

**Published:** 2026-03-25

**Authors:** Yantao Zhang, Zhenxing Zhu, Piyao Ji, Jianghua Ming, Yan Zhou

**Affiliations:** 1 Department of Orthopedics, Renmin Hospital of Wuhan University, Wuhan, China; 2 Central Laboratory, Renmin Hospital of Wuhan University, Wuhan, China

**Keywords:** Checkpoint signalling, Cytokinesis

## Abstract

Mitochondrial dysfunction-driven senescence is a central mechanism in the development of osteoarthritis (OA). Leucine-rich repeat kinase 2 (LRRK2), a multifunctional kinase implicated in maintaining mitochondrial homeostasis, has been examined in several inflammatory conditions. However, its role in regulating cellular senescence and its pathogenic contribution to OA remain insufficiently understood. To clarify the mechanism by which LRRK2 contributes to OA, RNA-seq and bioinformatics analysis were performed, followed by in vivo validation using a destabilization of medial meniscus (DMM) rat model in which LRRK2 was overexpressed *via* recombinant adeno-associated virus (rAAV). Complementary in vitro experiments were carried out to assess the impact of LRRK2 on mitochondrial dysfunction and senescence in chondrocytes. Our posttranscriptional analyses identified regulated factor influencing OA-related gene expression and revealed a strong association between LRRK2 and senescence-related regulatory genes in OA. rAAV-mediated LRRK2 overexpression accelerated chondrocyte senescence and worsened cartilage degeneration in DMM-induced OA. LRRK2 promoted HMGB1 upregulation by modulating GTPase activity, aggravating chondrocyte senescence. LRRK2 activated the cGAS–STING signaling pathway, increasing HMGB1 expression, exacerbating cellular senescence, and intensifying mitochondrial dysfunction. Treatment with the STING inhibitor H-151 partially mitigated the LRRK2-induced enhancement of chondrocyte senescence and mitochondrial impairment. This study demonstrates that LRRK2 drives chondrocyte senescence in OA by activating the cGAS–STING–HMGB1 axis, highlighting LRRK2 as a potential therapeutic target for OA.

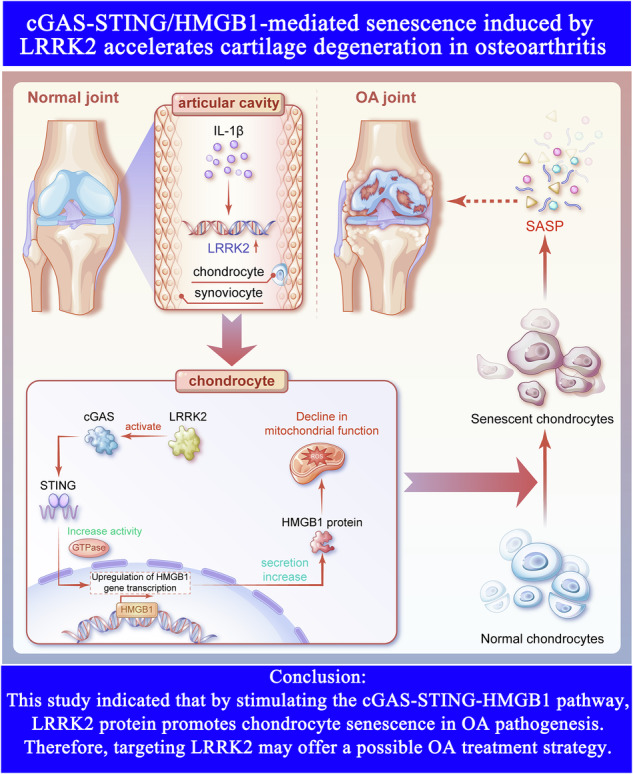

## Introduction

Osteoarthritis (OA) is a chronic degenerative joint disorder characterized by progressive breakdown of the articular cartilage matrix. Its primary pathological features include subchondral bone hyperplasia, synovial inflammation, and cartilage degradation [1, 2]. Evidence indicates that multiple senescence-related factors drive OA in older adults. Oxidative stress, reduced autophagy, and mitochondrial dysfunction contribute to aberrant functional changes in chondrocytes, including senescence, apoptosis, and impaired tissue maintenance [3, 4]. Although several therapeutic agents are currently under clinical evaluation, no treatment has been shown to halt or reverse OA progression [5], likely due to the substantial pathological and molecular heterogeneity of the disease [6].

Despite phenotypic variability across OA subtypes, shared pathogenic mechanisms, particularly chondrocyte senescence, are consistently observed [7, 8]. Cellular senescence is a fundamental feature of aging, and chondrocytes demonstrate multiple senescent characteristics as OA advances [9]. Senescent cells adopt a senescence-associated secretory phenotype (SASP), demonstrate reduced proliferative capacity, and undergo increased apoptosis, while altering the morphology and function of surrounding cells and tissues [10]. SASP is enriched in proinflammatory mediators, including matrix metalloproteinase-3 (MMP-3), tumor necrosis factor-α (TNF-α), interleukin (IL)-1, and IL-6, mirroring the inflammatory processes involved in OA onset [11]. Various studies have demonstrated that chondrocyte senescence is a major driver of OA pathogenesis; however, the precise mechanisms governing this process remain incompletely understood [12, 13].

**Fig. 1:**
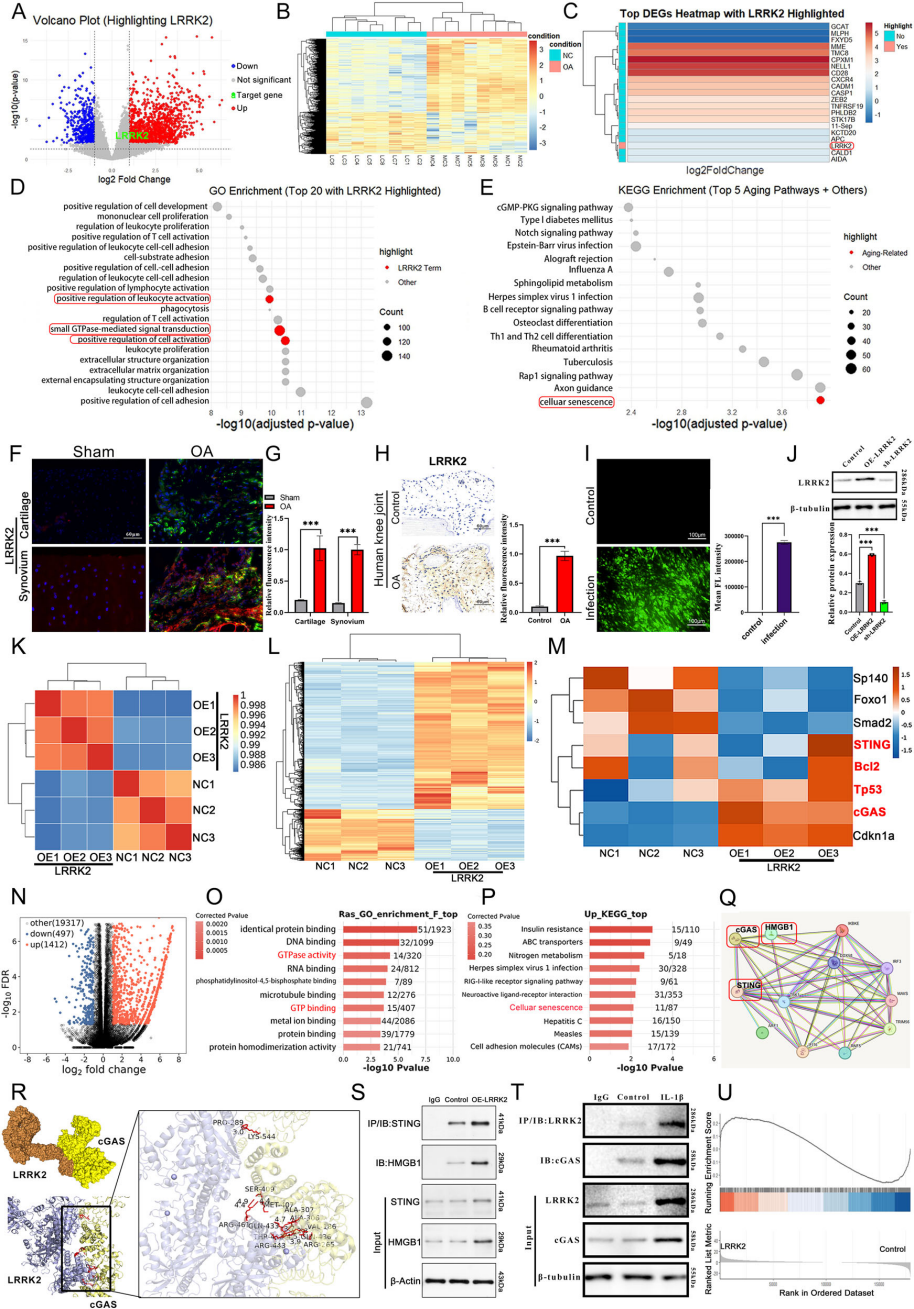
Effects of LRRK2 overexpression on the gene expression profile of chondrocytes. **A** GEO analysis of significantly differentially expressed genes in OA patients and highlighted LRRK2 indicated by volcano plot. **B** The heatmap showed the gene expression patterns of NC versus OA humans. **C** The heat map showed significantly differentially expressed genes, in which LRRK2 was highlighted. **D** GO analysis of factors highly expressed in OA patients and LRRK2 was highlighted. **E** KEGG enrichment analysis was used to analyze the pathways related to LRRK2. **F**, **G** Immunohistochemical results of cartilage and synovial cells in rats, histogram showed that the OA group was significantly higher than the sham group. **H** Carrying out immunohistochemical detection and statistical analysis on the normal and OA articular cartilage of human knees. **I** Successful transfection was demonstrated by fluorescent expression of chondrocytes after LRRK2 transfection using lentivirus. **J** Western blotting detection and statistical analysis on overexpression and knockdown of LRRK2. **K** Cluster analysis of the samples showed a high degree of similarity, which confirmed the correctness of the experimental design and sample sampling. **L** The heatmap showed that the gene expression patterns and clustering relationships of the samples were similar. **M** The enrichment heatmap of cellular senescence pathways showed that many genes, including STING, cGAS, BCL2, P53, and Cdkn1a, were closely related to cell senescence. **N** Comparison between samples showed the number of differentially expressed genes. **O** GO enrichment analysis was used to analyze the function of related proteins after LRRK2 overexpression. **P** KEGG enrichment analysis was used to analyze the senescence-related pathways after LRRK2 overexpression. **Q** The connections among cGAS, STING, and HMGB1 in the protein interaction network was analyzed. **R** The predicted model diagram of molecular docking between LRRK2 and cGAS, along with the amino acids at the hydrogen bond binding sites. **S** The Co-IP method was used to detect the binding situation between LRRK2 and cGAS. **T** The Co-IP experiment was conducted on the control group and the IL-1β group. Under the senescence condition, the binding of LRRK2 to cGAS increased. **U** GSEA enrichment analysis of senescence-related gene expression. Data were presented as mean ± SD (*n* = 3). Statistical analysis was performed using Student’s *t* test, ^***^p < 0.001.

Cellular senescence is closely linked to metabolic decline and mitochondrial dysfunction [14]. Using publicly available datasets and RNA sequencing, our early bioinformatics analyses identified leucine-rich repeat kinase 2 (LRRK2) as a significantly dysregulated and highly enriched senescence-related gene in OA (Fig. 1A, C, E). LRRK2 is a large multidomain protein of more than 2500 amino acids, comprising ankyrin repeats, leucine-rich repeats, a ROC GTPase domain, a C-terminal ROC (COR) region, a MAPK-related kinase domain, and a WD40 domain [15]. Mutations in LRRK2 are the most frequent genetic cause of familial Parkinson’s disease [16], and have been linked to increased susceptibility to inflammatory disorders such as inflammatory bowel disease, tuberculosis, and leprosy, underscoring its importance in inflammatory regulation [17]. A recent work demonstrates that LRRK2 overexpression disrupts mitochondrial homeostasis in macrophages, reprograms cell death pathways, and substantially elevates intracellular reactive oxygen species (ROS) levels [18]. However, its involvement in OA has not been explored. Based on these observations, it is hypothesized that LRRK2 may promote chondrocyte senescence by aggravating mitochondrial dysfunction, ultimately contributing to irreversible joint damage.

Under physiological conditions, DNA is localized to the nucleus and mitochondria [19]. Cytoplasmic DNA is sensed by the cyclic GMP–AMP synthase (cGAS)–stimulator of interferon genes (STING) inflammatory pathway [19]. Our preliminary Ras ratio thermal analysis showed that LRRK2 overexpression significantly upregulated genes associated with the cGAS–STING pathway (Fig. 1M). Previous research has established a key role for the cGAS–STING pathway in cellular senescence [20], and STING activation has been implicated in the initiation of OA and promotion of chondrocyte senescence in mice [21]. High mobility group box 1 (HMGB1), a non-histone chromatin-binding protein and damage-associated molecular pattern (DAMP), induces p53-dependent cellular senescence [22]. Recent findings indicate that cGAS–STING activation elevates plasma HMGB1 levels [18]. Whether LRRK2 regulates the cGAS–STING–HMGB1 axis to modulate chondrocyte senescence, however, remains unclear.

This study aims to clarify the relationship between LRRK2 and OA onset and to elucidate the mechanism by which LRRK2 drives chondrocyte senescence. The role of LRRK2 in OA development and progression and its underlying regulatory pathways were investigated. The study suggests that LRRK2 promotes OA-related chondrocyte senescence through activation of the cGAS–STING–HMGB1 signaling axis. To our knowledge, this is the first report demonstrating the detrimental role of LRRK2 in OA pathogenesis and chondrocyte senescence. These findings provide new evidence linking LRRK2 to OA progression and highlight its potential as a therapeutic target.

## Methods and materials

### Reagents and antibodies

LRRK2 antibody (#A17253) was obtained from Abclonal Antibody (Wuhan, China). Anti-HMGB1 (#CPA7037) was obtained from Cohesion Biosciences (London, UK). Anti-P53 (#21891-1-ap), TOM20 (#11802-1-ap), SOD2 (#24127-1-ap), MMP-3 (#17873-1-ap), Bcell lymphoma-2 (Bcl-2) (#26593-1-ap), TMEM173/STING (#19851-1-ap), P21 (#10355-1-ap), cGAS (#26416-1-ap), IL-6 (#21865-1-ap), TNF-α (#29652-1-ap), Vimentin (#10366-1-ap), and P16INK4A (#10883-1-ap) were purchased from Proteintech Group, Inc (Wuhan, China). β-tubulin (#T0023) was obtained from Affinity Biosciences (Cincinnati, OH, USA). Mitochondrial membrane potential assay kit with Rhodamine-123 and JC-1, Cell Couting Kit-8 (CCK-8), MitoTracker Red CMXRos, ROS Assay Kit, Hoechst 33342 Kit, and SA-β-galactosidase staining (SA-β-gal) assay kit were purchased from Beyotime Biotechnology (Shanghai, China). Fetal bovine serum (FBS) and penicillin/streptomycin were obtained from Gibco BRL (Maryland, USA). LRRK2 inhibitor IKK16, GTPase inhibitor ML141, and STING inhibitor H-151, was purchased from MCE Inc (New Jersey, USA). All other reagents were of analytical grade.

### RNA-sequencing and bioinformatics analysis

This bioinformatics analysis included data from the human gene expression database GSE286154. Furthermore, the standardized gene expression data of GSE286154 were obtained from the Gene Expression Omnibus (GEO) database. Then, the limma R tool was employed to search for differentially expressed genes (DEGs). The screening criterion was set as |log2 FoldChange|> 1 and *p*-value < 0.05. Subsequently, DEGs were subjected to Gene Ontology (GO) functional enrichment analysis *via* the cluster Profiler R package to identify the biological significance of genes. Moreover, the Kyoto Encyclopedia of Genes and Genomes (KEGG) signal pathway enrichment analysis was carried out to identify significantly enriched metabolic and signal transduction pathways. The Benjamini-Hochberg technique was used to compensate for multiple tests, and *p*.adjust < 0.05 was established as the significance criterion for GO and KEGG analyses. The acquired enrichment data were shown using the ggplot2 R package. The relevance of the gene distribution and enrichment roots was visualized by histograms, heat maps, volcano plots, and bubble maps.

The RNA samples were employed with sufficient volume and reduced heterogeneity for RNA sequencing using an Illumina HiSeq 4000. In total, 6 transcriptome sequencing samples, including Over-Expression (OE)_LRRK2_1st, OE_LRRK2_2nd, OE_LRRK2_3rd, NC_1st, NC_2nd, and NC_3rd, were acquired. The expression of a particular gene was denoted by the Per kilobase transcript per million map reads (FPKM). An Agilent 2100 bioanalyzer (LABX, Midland, Canada) verified the RNA marker. For RNA sequencing, “RNA compatibility number” 6.5 was used as the template. Low-quality environmental pollutants and high-pollution compounds with a high quantity of base (N) noise were filtered using the gene expression profile. Then, Bowtie244 was employed to compare the profile with the reference gene values (NCBI Rnor6.0). The DEGs were identified *via* DESeq2. For GO and KEGG enrichment analyses, as well as heatmap and volcano map acquisition, R Studio was utilized.

### RNA sequencing and gene set enrichment analysis (GSEA)

Gene set enrichment analysis (GSEA) was conducted using the ClusterProfiler package (v4.4) in R. Genes were ranked according to the signal-to-noise ratio based on log2 fold change values, and enrichment was assessed against the Hallmark, KEGG, and GO gene sets from the Molecular Signatures Database (MSigDB v7.5.1). To specifically interrogate aging-related processes, we curated senescence-associated signatures (including cellular senescence, DNA damage response, SASP, and telomere maintenance pathways). Enrichment scores were calculated using 1000 permutations, and significance was defined as a false discovery rate (FDR) < 0.25 following GSEA convention. Results were visualized with enrichment plots and normalized enrichment scores (NES) to highlight key senescence-associated pathways.

### Molecular docking

The crystal structures of LRRK2 (PDB: 8vh4) and cGAS (PDB: 7c0m) were obtained from the RCSB Protein Data Bank (www.rcsb.org). Structural preparation was performed using AutoDockTools, including removal of water molecules, addition of hydrogen atoms, and conversion to pdbqt receptor files. Molecular docking simulations and binding energy calculations were conducted using AutoDock Vina. The resulting protein–ligand complexes were visualized and evaluated for binding interactions and affinity with PyMOL (http://www.pymol.org/pymol) and Discovery Studio.

### Animal experiment

Male Sprague–Dawley (SD) rats (6–8 weeks old, 160–180 g) were purchased from Hunan SJA Laboratory Animal Co., Ltd. (Changsha, China). Following a one-week acclimation period, animals were randomly assigned to five groups: sham operation, OA induction, rAAV-LRRK2 + OA, OA + control rAAV (con-rAAV), and OA + rAAV-LRRK2 + H-151 (*n* = 6 per group). Simple randomization was used to minimize allocation bias. For OA induction, rats were anesthetized with inhaled isoflurane (2–5% in 100% oxygen), and osteoarthritis was induced in the right knee by transecting the medial meniscus to create the destabilization of the medial meniscus (DMM) model.

In the rAAV-LRRK2 group, animals received weekly intra-articular injections into the right knee consisting of rAAV encoding LRRK2 (Shanghai Gene-chem Co., Ltd.; 1 × 10¹⁰ dRP/30 μL per joint) and 10 μL phosphate-buffered saline (PBS), beginning 4 weeks after surgery. Rats in the OA + con-rAAV group received 1 × 10¹⁰ dRP/30 μL con-rAAV plus 10 μL PBS. The OA + rAAV-LRRK2 + H-151 group was administered 1 × 10¹⁰ dRP/30 μL rAAV-LRRK2, 10 μL PBS, and H-151 to achieve a final intra-articular concentration of 1 mM. Sham-operated and OA-only groups were injected with 40 μL PBS in the right knee. All animals were euthanized via cardiac exsanguination 10 weeks after surgery, and tissue samples were collected for further analysis.

The sample size (*n* = 6 per group) was based on preliminary data and previously published DMM-induced OA studies, which demonstrated that this group size provides sufficient statistical power (≥80%) to detect meaningful differences in histological outcomes, such as Mankin scores, at an alpha level of 0.05. No formal a priori power calculation was performed.

### Histological analysis

Each rat’s knee joint was dissected, fixed with 4% paraformaldehyde for 24 h, decalcified for 6 weeks, and then embedded in paraffin. Then, sagittal sections (5 µm) of the knee were prepared and stained with hematoxylin-eosin (H&E) and Safranin O stains. The two observers conducted the pathophysiological study in a double-blind manner, and the modified Mankin scoring system was employed [23].

### In vivo micro-MRI

The sagittal location of the femur’s medial condyle was selected from each group to quantify the water signals of these bones *via* the ImageJ software (National Institutes of Health, Bethesda, MD). Each rat’s right knee joint was assessed using a 9.4 T high magnetic field micro-MRI (BioSpec 70/30 USR, Germany). Furthermore, to confirm possible bone marrow lesion-/edema-like events, fat suppression, spin-echo, and T2-weighted scan modalities were employed.

### Micro-CT examination

Knee joints were scanned using a micro-CT system (Bruker MicroCT N.V., SkyScan 1176, Kontich, Belgium) at a resolution of 4000 × 2672 pixels with a voxel size of 9 μm. Following detailed image processing, three-dimensional morphometric analysis was performed to evaluate bone volume fraction (BV/TV), trabecular thickness (Tb.Th), and trabecular separation (Tb.Sp).

### Immunohistochemistry, immunofluorescence and co-localization analysis

Immunohistochemical staining was performed to assess streptavidin–peroxidase labeling and quantify the proportion of positively stained cells. Tissue sections were processed using standard procedures and examined under a light microscope. The percentages of immunofluorescence-positive cells for LRRK2, cGAS, STING, MMP-3, P16INK4A, and HMGB1 were quantified using Image-Pro Plus 6.0 (Media Cybernetics, USA). For immunofluorescence detection, samples were incubated with fluorescence-conjugated secondary antibodies in the dark for 1 h and imaged using a fluorescence microscope (AX10, Carl Zeiss). Co-localization analysis of LRRK2 and cGAS in chondrocytes was performed using immunofluorescence staining, and Pearson’s correlation coefficient was calculated with the ImageJ Coloc2 plugin to quantify co-localization.

### Cell isolation, culture and stimulation

Primary rat knee joint cartilage was cultured using standard tissue culture techniques for 5 days. Cells were then digested with 0.25% trypsin + 0.02% EDTA for 1 h, seeded into plates containing 0.2% type II collagenase, and incubated at 37 °C for 4–5 h. Following centrifugation at 1000 rpm for 5 min, cells were resuspended in complete DMEM/F12 medium supplemented with 10% fetal bovine serum and penicillin/streptomycin. Third-passage chondrocytes were seeded into 6-well plates and allowed to adhere for 12 h before the medium was refreshed.

LRRK2 overexpression or silencing in chondrocytes was achieved using lentiviral transfection (GeneChem, Shanghai, China). For transduction, serum-free medium, HiTransG reagent, and 20 µL of lentivirus were added to each well and incubated at 37 °C for 16 h; the medium was then replaced with complete culture medium. After 72 h, infection efficiency was assessed using a fluorescence microscope, and fluorescence intensity was quantified to confirm successful transduction. Chondrocytes with LRRK2 overexpression were then used for subsequent experiments. For in vitro assays, treatments were randomly assigned to wells within each plate to minimize positional bias.

### Transmission electron microscopy (TEM)

For TEM, third-generation chondrocytes were cultured, trypsinized, collected, and fixed in 2.5% glutaraldehyde prepared in 0.1 M PBS (pH 7.3). Samples were then post-fixed with 1% osmium tetroxide at 4 °C, washed with distilled water after 3 h, dehydrated through a graded ethanol series, and embedded in epoxy resin. Ultrathin sections were examined using an H-600 IV TEM system (Hitachi, Japan). Nuclear morphology, mitochondrial integrity, and endoplasmic reticulum structure were assessed from the resulting images.

### SA-β-gal assay and senescence tracker staining

After 24 h of treatment, chondrocytes were fixed with 4% formaldehyde (Beyotime, China) for assessment of cell viability. Senescence-associated β-galactosidase (SA-β-gal) staining was performed using the SA-β-gal staining kit according to the manufacturer’s instructions. β-galactosidase activity was further evaluated using a senescence tracker probe (Beyotime, China), which was added to the culture medium at a 1:1000 dilution. Cells were incubated for 24 h and then examined under a fluorescence microscope (Olympus, Japan).

### Western blotting

Chondrocyte proteins were extracted using a whole protein extraction kit, separated by SDS-PAGE, and transferred onto PVDF membranes. Membranes were blocked in TBST containing 5% (w/v) skim milk for 1 h, then incubated overnight at 4 °C with primary antibodies against β-tubulin, LRRK2, cGAS, STING, HMGB1, P53, Bcl-2, MMP-3, TOM20, SOD2, TNF-α, IL-6, P21, and P16. TBST consisted of Tris-HCl (1 M, pH 7.5), NaCl (8 g), KCl (0.2 g), and Tween-20 (0.5 mL) in 1 L of distilled water. After primary incubation, membranes were treated with HRP-conjugated secondary antibodies for 1 hour and developed using an electrochemiluminescence (ECL) detection reagent (Amersham Biosciences, USA). Signals were visualized and analyzed using the Odyssey infrared imaging system (LI-COR Biosciences, NE, UK). Target protein expression levels were normalized to the intensity of the β-tubulin band.

### Mitochondrial membrane potential assay

Mitochondrial membrane potential was assessed using the JC-1 and Rhodamine-123 assay kits. Chondrocytes were incubated with either 800 μL of JC-1 working solution or 10 μM Rhodamine-123 at 37 °C for 25 min. After the staining solution was removed, cells were cultured in 2 mL of complete DMEM/F12 supplemented with Hoechst. Red and green fluorescence intensities, as well as the red/green fluorescence ratio, were subsequently measured to evaluate mitochondrial membrane potential.

### MitoTracker Red staining

Chondrocytes were labeled with MitoTracker Red, washed with PBS, and then incubated with a 50 nM MitoTracker Red working solution for 20 min at 37 °C. After staining, the solution was removed and replaced with fresh culture medium. Mitochondrial morphology was then examined using a fluorescence microscope (Olympus, Japan).

### ROS level analysis

The ROS levels were measured using a ROS assay kit. Cells were incubated with diluted DCFH-DA (1:1000) at 37 °C for 20 min. ROS production was then quantified using a fluorescence microscope (Olympus, Japan) and a flow cytometer (Beckman Coulter, USA).

### Mitochondrial ATP measurement

Mitochondrial ATP levels were assessed using a mitochondrial ATP assay kit in combination with Hoechst staining. Chondrocytes were first washed and then transfected with a pCMV-Mito-AT plasmid (Beyotime, China) in 1 mL of complete DMEM/F12 medium at 37 °C for 4 h. After removing the transfection solution, cells were incubated in 2 mL of fresh complete DMEM/F12 medium. Green fluorescence signals were subsequently visualized using a fluorescence microscope (Olympus, Japan), and fluorescence intensity was quantified to determine mitochondrial ATP content.

### Detection of ROS levels through flow cytometry

Chondrocytes were dissociated with 0.25% trypsin-EDTA, centrifuged at 300 × *g* for 5 min, and resuspended in 500 μL PBS for flow cytometric analysis. Intracellular ROS levels were quantified by measuring the mean fluorescence intensity of DCFH-DA using a flow cytometer (Beckman Coulter, USA) with excitation at 488 nm and emission at 525 nm. Approximately 10,000 events were recorded per sample, and data were analyzed using FlowJo software (Tree Star, USA).

### Detection of chondrocyte viability through CCK-8 assay

Chondrocytes (1000 cells/well) were seeded into 96-well plates and maintained at 37 °C with 5% CO_2_ until reaching 70–80% confluence. Cell viability was evaluated using the CCK-8 assay. Following treatment, 10 μL of CCK-8 solution was added to each well and incubated for 2 h at 37 °C in the dark. Absorbance at 450 nm was then measured using a BioTek Synergy H1 microplate reader. Each experiment included *n* = 5 independent replicates, a sample size deemed adequate to detect expected differences based on prior assay optimization.

### Co-immunoprecipitation (Co-IP)

Cells were lysed in ice-cold lysis buffer (50 mM Tris-HCl, pH 7.5; 150 mM NaCl; 1% NP-40; and protease/phosphatase inhibitor cocktails) for 30 minutes on ice, then centrifuged at 12,000 × *g* for 15 min at 4 °C to remove debris. The resulting supernatants were incubated overnight at 4 °C with the specified primary antibodies, with gentle rotation. Protein A/G agarose beads (MCE, USA) were then added and incubated for 2–4 h at 4 °C. Beads were washed three times with lysis buffer to reduce nonspecific binding, and bound proteins were eluted by boiling in SDS loading buffer for 5 min. Samples were analyzed by SDS–PAGE followed by immunoblotting with the appropriate antibodies. Normal IgG served as a negative control to exclude nonspecific interactions.

### Blinding

In the in vivo experiments, the investigators who performed the DMM surgeries, administered injections, and provided postoperative care were necessarily aware of group assignments due to the nature of the procedures. However, outcome evaluations were conducted in a blinded manner whenever feasible. Personnel responsible for histological scoring, micro-MRI and micro-CT image analysis, and quantitative molecular and cellular assessments, including western blot densitometry, immunofluorescence quantification, and flow cytometry, were blinded to sample identities. For in vitro studies, treatment groups were coded, and investigators conducting the experiments and analyzing the data remained blinded to group assignments until all analyses were completed.

### Statistical analysis

For comparisons involving three or more groups, one-way ANOVA followed by Bonferroni post hoc correction was performed. Data are reported as mean ± standard deviation (SD), and *p* < 0.05 was considered statistically significant. For experiments involving specimens from three to five donors, independent *t*-tests were conducted to assess group differences.

### Sample inclusion and exclusion criteria

In the animal studies, rats were excluded if they died before the planned endpoint, showed severe postoperative infection, or showed evidence of surgical failure (e.g., incorrect ligament transection). These criteria were defined before data collection, and no animals met the exclusion criteria. For in vitro experiments, cell cultures were excluded if contamination occurred, transfection efficiency fell below 70% (as determined by parallel fluorescence controls), or cell mortality exceeded 50% under control conditions. All exclusion criteria were predetermined.

## Results

### LRRK2 was significantly elevated in human and rat OA models

A comprehensive bioinformatics analysis of the publicly available GEO dataset GSE286154, which includes cartilage tissue samples from eight OA patients and eight healthy controls, was performed to investigate transcriptional alterations associated with OA. Raw expression data were normalized using the limma package in R, and principal component analysis was applied to correct batch effects. Differential expression analysis identified 1243 significantly altered genes in OA (adjusted *p* < 0.05, |log₂ fold change|> 1). A volcano plot demonstrated that LRRK2 was among the most significantly dysregulated genes (Fig. 1A).

To visualize global expression patterns, hierarchical clustering of DEGs was performed and presented as a heatmap (Fig. 1B). OA and control samples segregated into distinct clusters, with LRRK2 showing significant differential expression (Fig. 1C). Gene Ontology (GO) enrichment analysis revealed significant upregulation of pathways associated with LRRK2 activity, such as “positive regulation of leukocyte activation”, “small GTPase-mediated signal transduction”, and “positive regulation of cell activation”, in OA samples (FDR < 0.05) (Fig. 1D). KEGG pathway enrichment further indicated activation of senescence-related pathways in OA cartilage (Fig. 1E).

A DMM-induced rat OA model (*n* = 6) was then established to validate these findings, and LRRK2 expression was compared in cartilage and synovial tissue (Vimentin-labeled) with that of sham-operated controls (*n* = 6). Immunofluorescence staining revealed robust cytoplasmic overexpression of LRRK2 in OA rats. In comparison, sham animals showed minimal staining (Fig. 1F). Quantitative analysis showed that LRRK2 expression in OA samples was approximately threefold higher than in sham controls (Fig. 1G). Similarly, immunohistochemical staining of human knee cartilage demonstrated significantly elevated LRRK2 expression in OA patients relative to normal cartilage (Fig. 1H). These transcriptional and protein-level findings confirm that LRRK2 is significantly upregulated in OA and support its central involvement in OA pathogenesis.

### LRRK2 was significantly correlated with senescence

A chondrocyte LRRK2 overexpression model was successfully generated using lentiviral transduction. Fluorescence microscopy confirmed significantly elevated LRRK2 expression in the OE-LRRK2 group compared with controls, indicating efficient model establishment (Fig. 1I). Similarly, an siRNA-mediated LRRK2 knockdown model was constructed and validated by western blotting. As expected, LRRK2 protein levels were significantly increased in the OE-LRRK2 group and significantly reduced in the sh-LRRK2 group relative to controls (Fig. 1J). Principal component analysis demonstrated clear separation between OE-LRRK2 and control samples, with all three biological replicates clustering appropriately (Fig. 1K).

FPKM values for all expressed genes were used to construct six unsupervised hierarchical clustering association matrices. LRRK2 overexpression produced substantial alterations in the chondrocyte transcriptome, with DEG clustering and sample grouping showing strong concordance. Heatmaps of FPKM values further highlighted pronounced differences in gene expression between groups (Fig. 1L).

Ras ratio thermal analysis revealed significant upregulation of senescence-associated and cGAS–STING pathway–related genes, including Bcl-2, P53, and P16, in the OE-LRRK2 group. These findings suggest that LRRK2 influences cellular senescence by activating the cGAS–STING axis (Fig. 1M). Differential expression analysis identified 1,412 upregulated and 497 downregulated genes following LRRK2 overexpression (Fig. 1N). GO enrichment analysis showed that upregulated genes were enriched in molecular functions related to GTP binding and GTPase activity (Fig. 1O), while KEGG pathway enrichment indicated a strong association with senescence-related signaling pathways (Fig. 1P). These observations were consistent with protein–protein interaction network analyses (Fig. 1Q).

Molecular docking analysis predicted stable binding between LRRK2 and cGAS proteins (Fig. 1R). Co-immunoprecipitation further validated this interaction: IL-1β stimulation enhanced LRRK2–cGAS binding in chondrocytes (Fig. 1T), and LRRK2 overexpression increased the association between STING and HMGB1 (Fig. 1S). These results support the hypothesis that LRRK2 promotes cellular senescence by activating the cGAS–STING–HMGB1 pathway. GSEA enrichment analysis of RNA-seq data further corroborated the upregulation of senescence-associated gene expression (Fig. 1U). These molecular findings demonstrate that LRRK2 activation of the cGAS–STING–HMGB1 signaling pathway drives chondrocyte senescence.

### LRRK2 aggravated OA progression in the rat OA model

Building on the above findings, rAAV-mediated LRRK2 overexpression was used to evaluate the role of LRRK2 in OA onset and progression. A DMM-induced OA model combined with intra-articular rAAV-LRRK2 delivery was generated to assess the effects of LRRK2 on rat knee joints. Similarly, rats received intra-articular injections of rAAV-LRRK2 together with the STING inhibitor H-151 to investigate whether LRRK2-driven OA pathology is mediated through the cGAS–STING pathway.

**Fig. 2:**
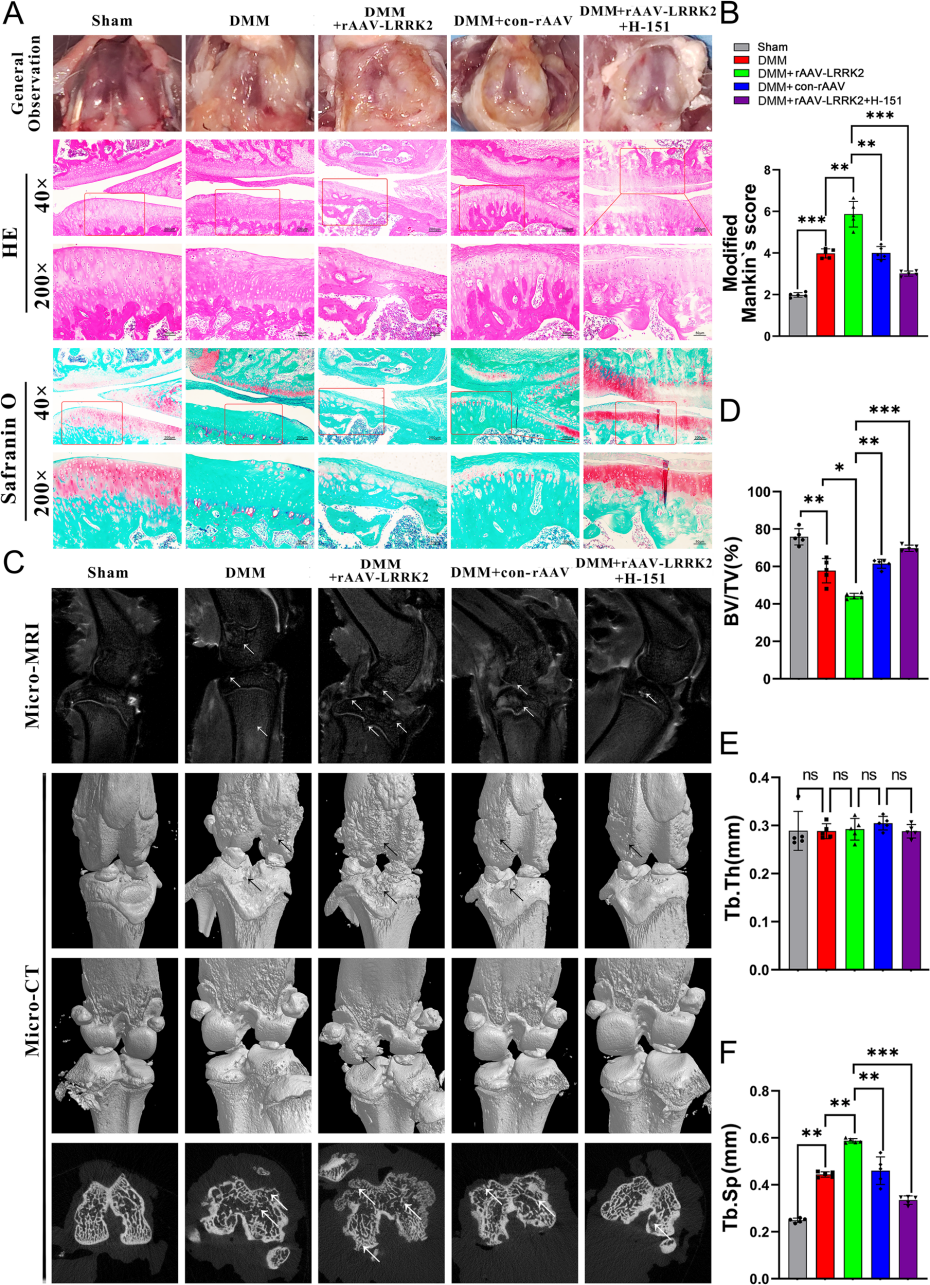
Regulation of LRRK2 on cartilage and subchondral bone in OA model rats. **A** General observations, H&E and Safranin O staining of rat articular cartilage at 10 weeks after surgery. **B** The modified Mankin scores were assigned to tissue samples (*n* = 6). **C** The pixel intensity and gross morphology of the sampling area of the epiphyseal trabeculae were evaluated by micro-MRI. White arrows indicate sites of cartilage damage and subchondral bone marrow edema. Representative micro-CT 3D reconstructions of the subchondral bone of the tibia and femur in the rats after DMM surgery. **D**–**F** Micro-CT images of animals in each group were used for measurements of BV/TV, Tb.Sp and Tb.Th (*n* = 5). Data were expressed as mean ± SD. Statistical analysis was performed using Student’s *t* test, ^*^p < 0.05; ^**^p < 0.01; ^***^p < 0.001.

Safranin O/Fast green staining revealed that LRRK2 overexpression significantly impaired the cartilage layer of the rat knee joint, whereas inhibition of the cGAS–STING pathway by H-151 partially restored cartilage integrity. Gross morphology and H&E staining further demonstrated extensive cartilage degeneration in the LRRK2 overexpression group, while H-151 treatment attenuated OA progression (Fig. 2A, B).

Micro-MRI imaging of the subchondral T2 signal (Fig. 2C) showed that sham-operated rats demonstrated clear, thin T2 signals corresponding to normal metaphyseal growth plate and cartilage structure. In comparison, the DMM + rAAV-LRRK2 group displayed significant subchondral bone and joint surface damage, reduced cartilage thickness, and increased bone degradation. H-151 administration mitigated these abnormalities. Micro-CT imaging also revealed joint cavity calcification, altered bone morphology, and osteophyte formation in the LRRK2-overexpressing group (Fig. 2C).

Quantitative micro-CT analysis indicated that, relative to sham animals, the DMM + rAAV-LRRK2 group showed a significant decrease in bone volume fraction (BV/TV) (Fig. 2D), an increase in trabecular separation (Tb.Sp) (Fig. 2F), and no significant change in trabecular thickness (Tb.Th) (Fig. 2E). These pathological changes were attenuated following H-151 treatment, further confirming the involvement of the cGAS–STING pathway in LRRK2-mediated OA deterioration.

### LRRK2 aggravated chondrocyte senescence and activated cGAS-STING in vivo

**Fig. 3:**
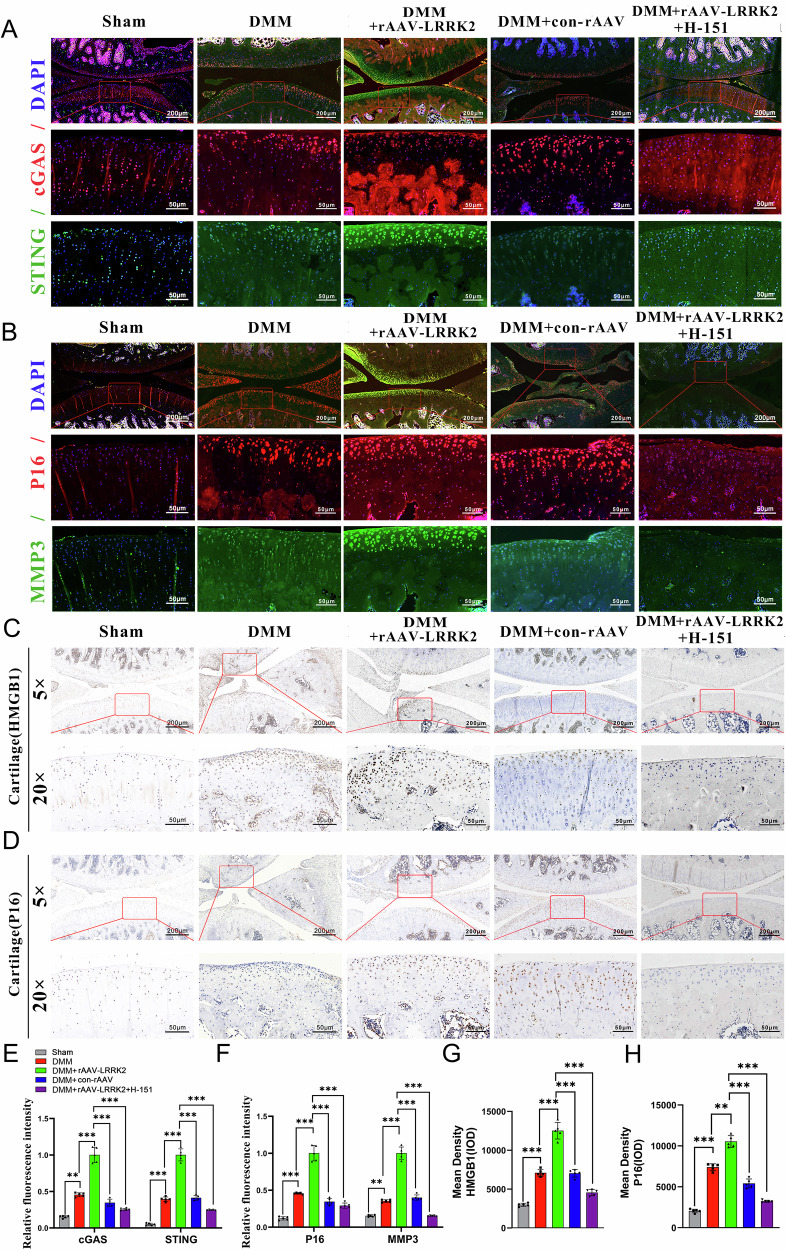
LRRK2 promoted chondrocyte senescence and catabolism in vivo in OA. **A**, **B** The immunofluorescence of cGAS, STING, MMP-3, and P16 were analyzed in the articular cartilage of OA rats induced by DMM receiving rAAV-OE-LRRK2. **C**, **D** Immunohistochemical detection of the expressions of HMGB1 and P16 in the knee joints of rats. **E**, **F** The cGAS, STING, P16, and MMP-3 proportions in articular cartilage were quantified by immunofluorescence. **G**, **H** Statistical quantitative analysis of immunohistochemical staining in P16 and HMGB1. Data were expressed as mean ± SD (*n* = 5). Statistical analysis was performed using Student’s *t* test, ^**^p < 0.01; ^***^p < 0.001.

Immunofluorescence staining was performed to assess STING and cGAS expression in rat articular cartilage. The DMM + rAAV-LRRK2 group showed significantly elevated levels of both cGAS and STING compared with the sham and DMM-only groups. This increase was reversed following H-151 treatment, indicating that LRRK2 exacerbates OA by activating the cGAS–STING pathway (Fig. 3A, E). Immunofluorescence analysis of MMP-3 and P16 expression in knee cartilage revealed similar trends: LRRK2 overexpression significantly upregulated these senescence- and matrix-related markers, while H-151 administration attenuated their expression (Fig. 3B, F). Immunohistochemical staining further demonstrated that HMGB1 and P16 protein levels were substantially higher in the DMM + rAAV-LRRK2 group than in both the sham and DMM-only groups (Fig. 3C, D, G, H). The immunofluorescence and immunohistochemical findings show that LRRK2 activates the cGAS–STING pathway, increases the expression of the inflammatory mediator HMGB1, accelerates chondrocyte senescence, and promotes OA progression.

### LRRK2 promoted ROS-mediated senescence of chondrocyte

**Fig. 4:**
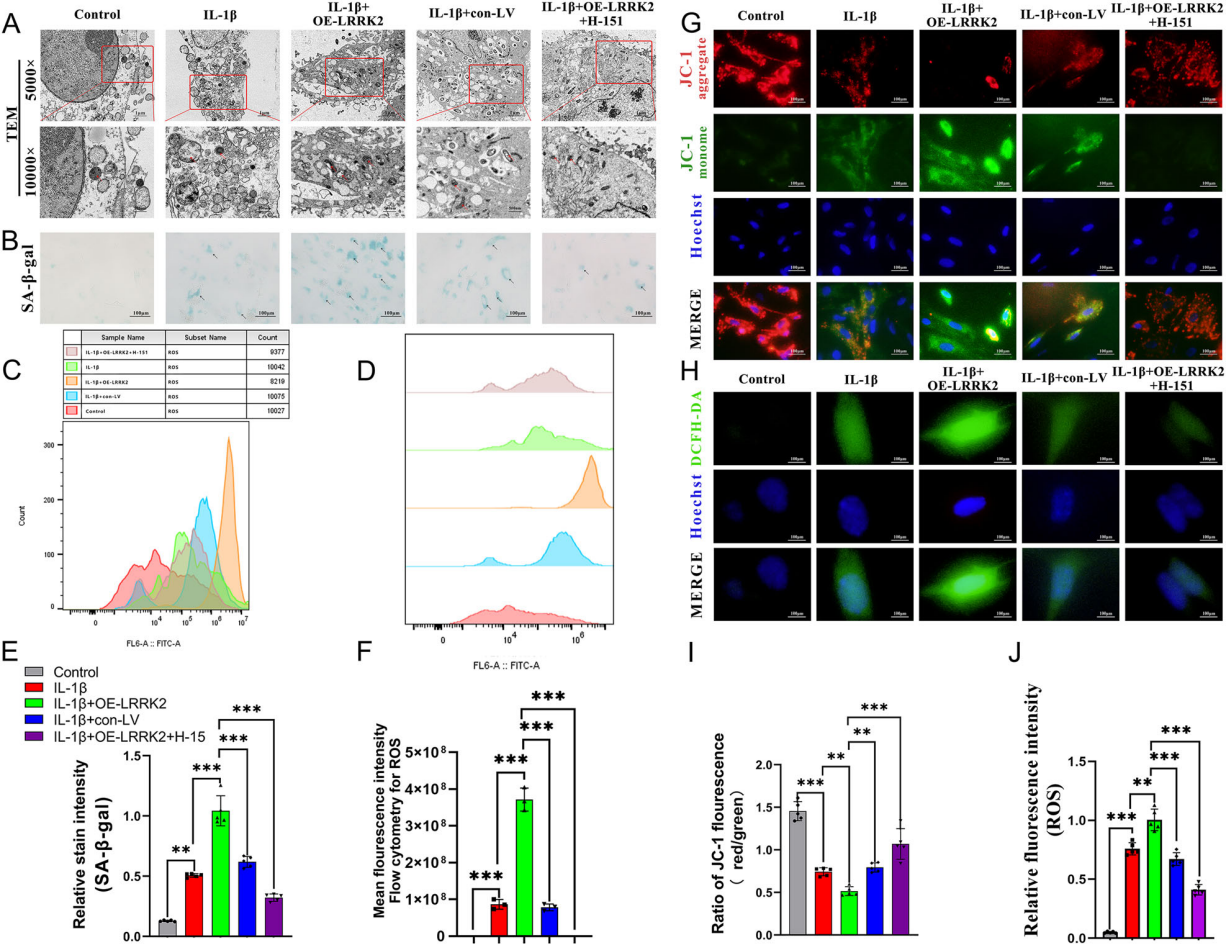
LRRK2-mediated chondrocyte senescence through ROS accumulation. **A** TEM showed that LRRK2 overexpression caused mitochondrial deformation and increased endoplasmic reticulum vacuolation. **B** SA-β-gal kit was used to detect the senescence of chondrocytes. **C**, **D** The ROS accumulation status of chondrocytes was examined by flow cytometry, and the mean fluorescence intensity was measured using FITC fluorescence channel. **E** Quantitative analysis of β-galactosidase staining of chondrocytes. **F** Quantitative analysis of ROS accumulation level. **G** Representative images of JC-1 staining. **H** DFCH-DA probe was used to detect the expression of ROS in chondrocytes. **I** The ratio of JC-1 fluorescence (red/green) was analyzed. **J** Quantitative analysis of relative ROS fluorescence intensity. Data were expressed as mean ± SD (*n* = 5). Statistical analysis was performed using Student’s *t* test, ^**^p < 0.01; ^***^p < 0.001.

To evaluate the role of LRRK2 in chondrocyte senescence, ultrastructural changes were examined using TEM. Control chondrocytes displayed intact nuclei, uniform chromatin distribution, and normal mitochondrial morphology (Fig. 4A). In comparison, IL-1β–treated cells demonstrated hallmark senescent features, including enlarged cytoplasmic vacuoles, dilated endoplasmic reticulum, and reduced mitochondrial abundance. The IL-1β + con-LV group showed similar changes to the IL-1β group, whereas these degenerative alterations were significantly intensified in the IL-1β + OE-LRRK2 group.

SA-β-gal staining further supported these findings. Compared with controls, IL-1β significantly increased the proportion of SA-β-gal–positive cells, and this effect was further amplified by LRRK2 overexpression (IL-1β + OE-LRRK2). Treatment with the STING inhibitor H-151 reduced the proportion of positively stained cells relative to the IL-1β + OE-LRRK2 group, indicating partial reversal of senescence (Fig. 4B, E).

To explore mechanisms underlying LRRK2-mediated senescence, intracellular ROS levels were assessed by flow cytometry using DCFH-DA. IL-1β elevated ROS production compared with controls, and LRRK2 overexpression further increased ROS levels (p < 0.01 vs. IL-1β) (Fig. 4C, D, F). Co-treatment with H-151 reduced ROS accumulation, demonstrating that LRRK2 promotes ROS generation by activating the cGAS–STING pathway. Fluorescence microscopy confirmed these findings: IL-1β–treated chondrocytes showed higher ROS signals than controls, OE-LRRK2 expression further enhanced ROS fluorescence, and H-151 treatment reduced both ROS production and fluorescence intensity (Fig. 4H, J). These results show that LRRK2 exacerbates oxidative stress and promotes chondrocyte senescence by activating the cGAS–STING pathway.

### LRRK2-mediated mitochondrial dysfunction led to chondrocyte senescence

**Fig. 5:**
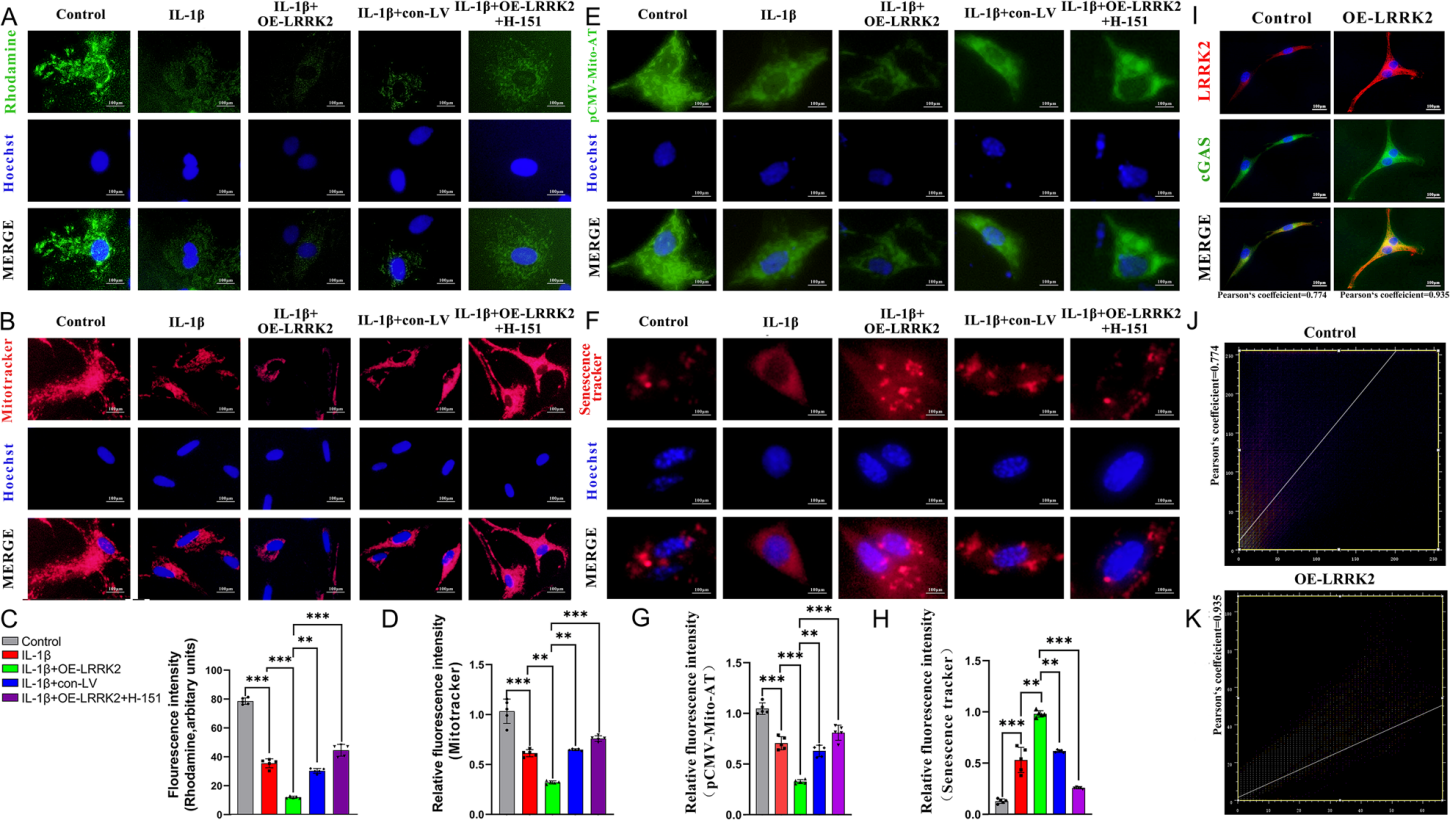
LRRK2-mediated mitochondrial dysfunction led to chondrocyte senescence. **A** Representative image of Rhodamine staining. **B** Mitochondrial viability was measured by Mitotracker representative fluorescence images. **C** Fluorescence quantitative analysis showed that LRRK2 overexpression significantly decreased mitochondrial viability in the IL-1β + OE-LRRK2 group compared with the IL-1β group. **D** Mitotracker fluorescence quantitative analysis showed that LRRK2 overexpression significantly reduced mitochondrial activity compared with IL-1β group. **E** The ATP content in mitochondria was detected using pCMV-Mito-AT. The fluorescence intensity was positively correlated with the ATP content. **F** Using the senescence tracker to detect β-galactosidase in chondrocytes, and the fluorescence intensity was positively correlated with the degree of senescence. **G** Fluorescence intensity of pCMV-Mito-AT staining. **H** The relative fluorescence intensity of the senescence tracker. **I** The immunofluorescence co-localization results of LRRK2 and cGAS proteins. **J** The immunofluorescence co-localization analysis of the control group using the “person’s coefficient”. **K** The immunofluorescence co-localization analysis of the OE-LRRK2 group using the “person’s coefficient”. Data were presented as mean ± SD (*n* = 5). Statistical analysis was performed using Student’s *t* test, ^**^p < 0.01; ^***^p < 0.001.

In chondrocytes treated with IL-1β and OE-LRRK2, JC-1 staining revealed significant mitochondrial depolarization, reflected by a reduced red/green fluorescence ratio compared with the control group, an effect attributable to LRRK2 overexpression. Inhibition of the cGAS–STING pathway with H-151 partially restored mitochondrial membrane potential, as evidenced by an increased red/green fluorescence ratio (Fig. 4G, I). Consistent with these findings, Rhodamine-123 (Fig. 5A, C) and MitoTracker (Fig. 5B, D) staining demonstrated that LRRK2 overexpression significantly decreased mitochondrial membrane potential, whereas H-151 treatment improved mitochondrial function.

Given the central role of mitochondrial dysfunction in cellular senescence, subsequent experiments explored how LRRK2-induced mitochondrial impairment contributes to senescence. Measurement of mitochondrial ATP content showed that ATP levels were significantly reduced following LRRK2 overexpression, while H-151 treatment effectively reversed this decline (Fig. 5E, G). Senescence-tracker assays further revealed a substantial increase in β-galactosidase activity in chondrocytes overexpressing LRRK2 (Fig. 5F, H). These results indicate that excessive LRRK2 expression severely compromises mitochondrial function through activation of the cGAS–STING pathway, ultimately accelerating chondrocyte senescence.

### LRRK2 enhanced the activity of GTPase to induce the expression of HMGB1

**Fig. 6:**
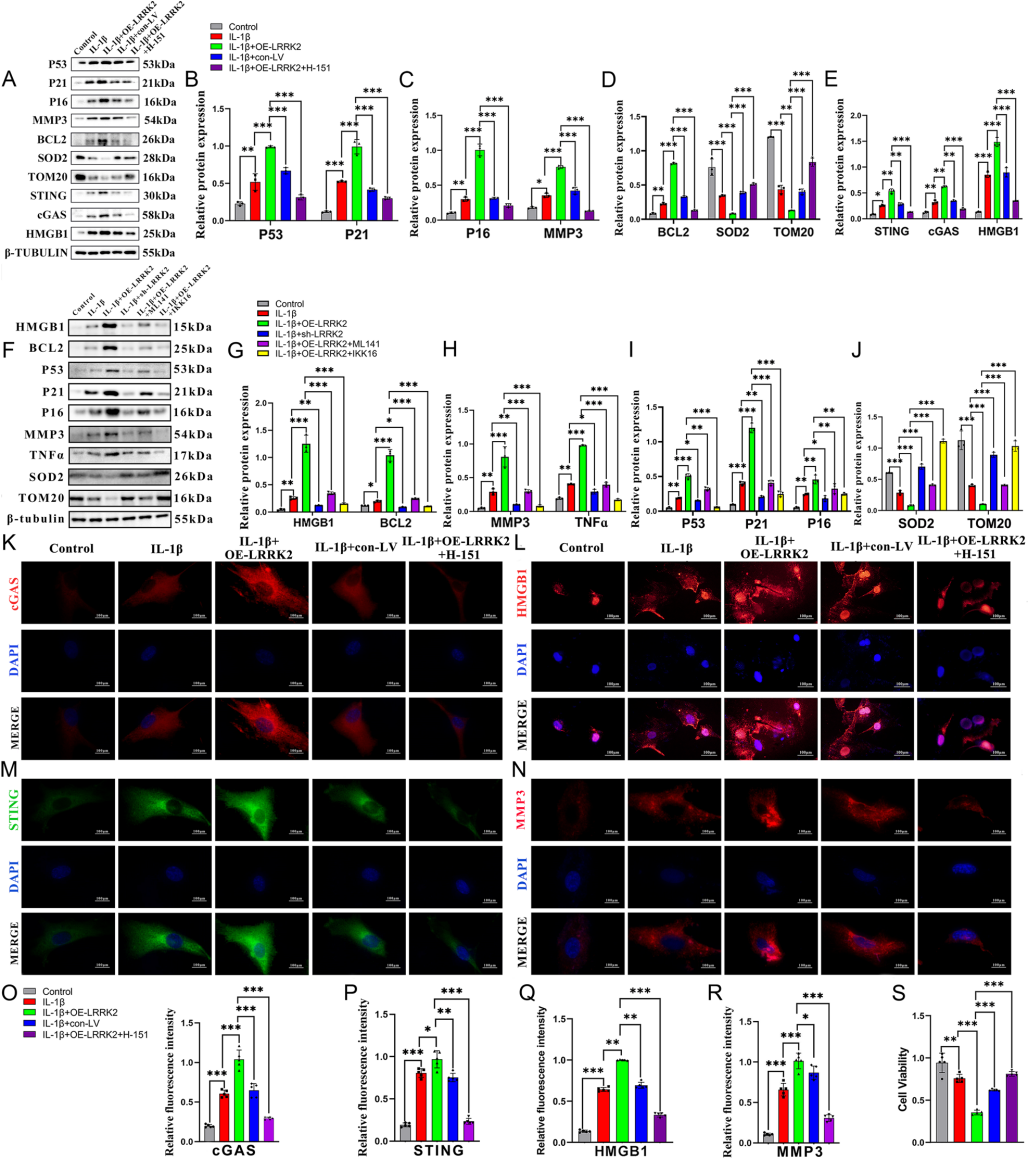
LRRK2 enhanced the activity of GTPase led to HMGB1 upregulation and chondrocyte senescence. **A** The protein expressions of P53, P21, P16, MMP-3, BCL2, SOD2, TOM20, STING, cGAS, and HMGB1 were assessed via western blotting with β-tubulin as a loading control. **B**–**E** Quantitative analysis of relative protein expression. **F** The protein expressions of P53, P21, P16, MMP-3, BCL2, SOD2, TOM20, TNF-α, IL-6, and HMGB1 were assessed using the GTPase activity inhibitor ML141 via western blotting. β-tubulin was used as the internal normalization processing. **G**–**J** Quantitative analysis of relative protein expression (*n* = 3). **K** Chondrocytes were stained by immunofluorescence with cGAS antibody and visualized using CY3 channels. **L** Chondrocytes were immunofluorescence stained with HMGB1antibody and visualized using CY3 channels. **M** Chondrocytes were immunofluorescence stained with STING antibody and visualized using FITC channels. **N** Chondrocytes were immunofluorescence stained with MMP-3 antibody and visualized using CY3 channels. **O** Quantitative analysis of cGAS immunofluorescence. **P** Quantitative analysis of STING immunofluorescence. **Q** The quantitative analysis of HMGB1 immunofluorescence. **R** Quantitative analysis of MMP-3 immunofluorescence. **S** Quantitative analysis of cell viability by CCK-8 assay. Data are presented as mean ± SD (*n* = 5). Statistical analysis was performed using Student’s *t* test, ^*^p < 0.05; ^**^p < 0.01; ^***^p < 0.001.

**Fig. 7:**
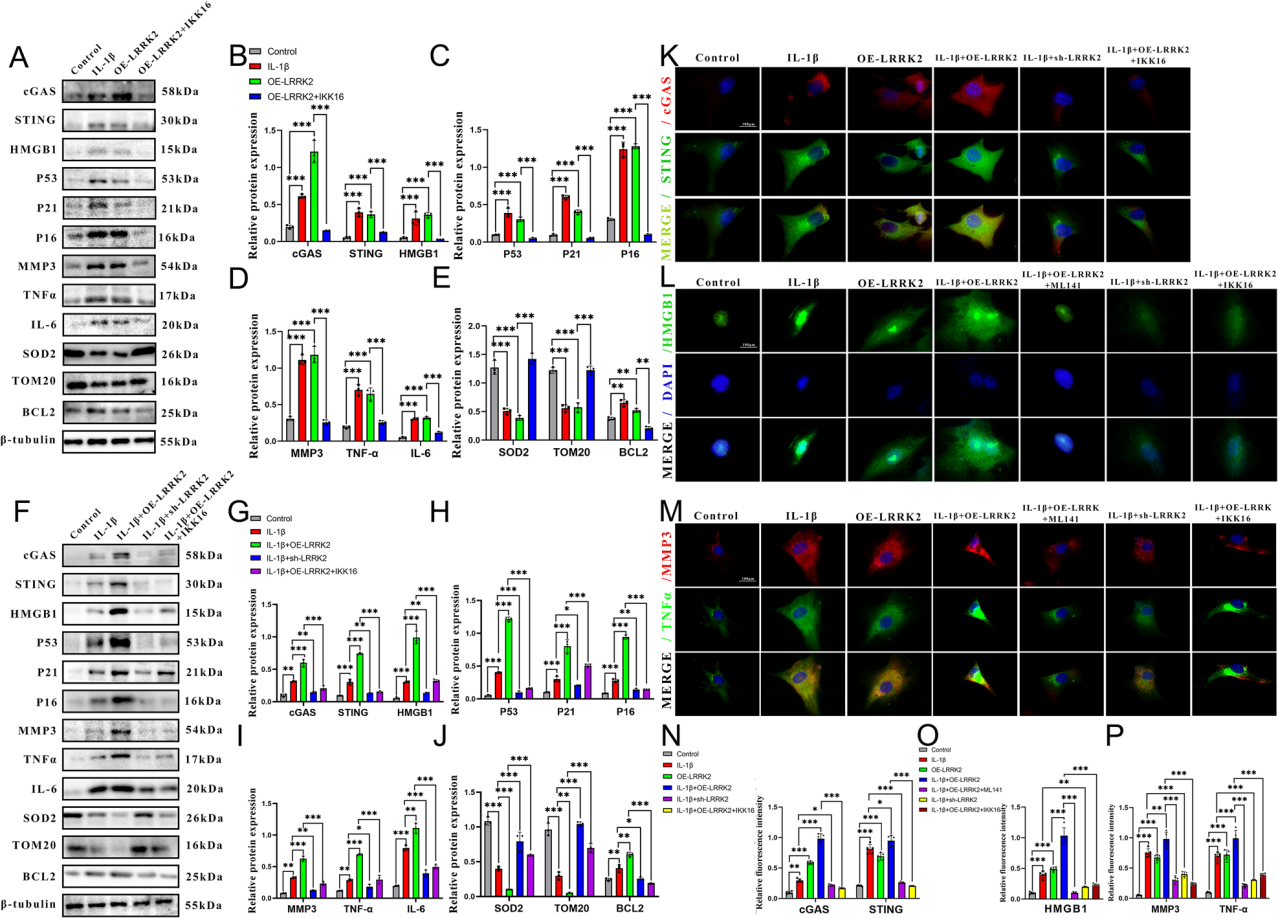
Overexpression of LRRK2 activated chondrocyte senescence by activating cGAS-STING/HMGB1 pathway. **A** The protein expressions of P53, P21, P16, MMP-3, BCL2, SOD2, TOM20, TNF-α, IL-6, STING, cGAS, and HMGB1 were assessed via western blotting. β-tubulin was used as the internal normalization processing. **B**–**E** Quantitative analysis of relative protein expression (*n* = 3). **F** The protein expressions of P53, P21, P16, MMP-3, BCL2, SOD2, TOM20, TNF-α, IL-6, STING, cGAS, and HMGB1 were assessed with LRRK2 silence via western blotting. β-tubulin was used as the internal normalization processing. **G**–**J** Quantitative analysis of relative protein expression (*n* = 3). **K** Immunofluorescence double-labeling analysis of cGAS and STING proteins. **L** Immunofluorescence analysis of HMGB1 expression and localization. **M** Immunofluorescence detection of MMP-3 and TNF-α expression. **N** Statistical analysis of cGAS and STING protein expression (*n* = 5). **O** Statistical analysis of HMGB1 protein expression (*n* = 5). **P** Statistical analysis of MMP-3 and TNF-α protein expression (*n* = 5). Statistical analysis was performed using Student’s *t* test, ^*^p < 0.05; ^**^p < 0.01; ^***^p < 0.001.

The mechanism through which LRRK2 regulates HMGB1 activation was also investigated. After inducing chondrocyte senescence with IL-1β, LRRK2 was overexpressed via lentiviral transduction and simultaneously treated with the GTPase inhibitor ML141 to determine whether LRRK2 influences HMGB1 expression through its GTPase activity. The results showed that ML141 treatment significantly reduced HMGB1 expression (Fig. 6F) and decreased downstream SASP factors, including TNF-α and IL-6, as well as other senescence-associated marker proteins (Fig. 6G–J). Treatment with the LRRK2 inhibitor IKK16 produced a similar reduction in protein expression (Fig. 6F). Immunofluorescence analysis further demonstrated that LRRK2 overexpression enhanced HMGB1 nuclear translocation and increased its cytoplasmic localization, whereas ML141 treatment reversed this effect (Fig. 7L, O). These findings indicate that LRRK2 promotes HMGB1 expression and cytoplasmic translocation by enhancing GTPase activity, amplifying inflammation, and accelerating chondrocyte senescence.

### LRRK2 activated chondrocyte senescence by activating the cGAS-STING-HMGB1 pathway

Western blotting and immunofluorescence analyses were performed to determine whether LRRK2 overexpression induces mitochondrial dysfunction through the cGAS–STING–HMGB1 pathway, driving cellular senescence and promoting OA progression. In the IL-1β–induced senescence model, LRRK2 overexpression significantly increased the levels of senescence markers, including P53, P21, P16, BCL2, and the SASP component MMP-3, relative to both the IL-1β and control groups (Fig. 6A–C). Concurrently, expression of mitochondrial activity–related proteins SOD2 and TOM20 was reduced (Fig. 6D). Proteins associated with the cGAS–STING–HMGB1 pathway were also substantially upregulated (Fig. 6E), supporting the conclusion that LRRK2 exerts its biological effects through activation of this pathway.

In the IL-1β + OE-LRRK2 + H-151 group, senescence-associated protein expression decreased, whereas mitochondrial activity proteins showed partial recovery. Both the STING inhibitor H-151 and the LRRK2 inhibitor IKK16 significantly downregulated cGAS, STING, and HMGB1 expression (Fig. 6K–Q). Immunofluorescence further demonstrated increased MMP-3 expression in the IL-1β + OE-LRRK2 group, a change mitigated by pathway inhibition (Fig. 6N, R). CCK-8 assays showed that LRRK2 overexpression impaired cell viability, an effect reversed by the addition of pathway inhibitors (Fig. 6S).

In both the LRRK2 overexpression model and the IL-1β-induced senescence model, proteins associated with the cGAS–STING–HMGB1 pathway, SASP (MMP-3, TNF-α, and IL-6), and senescence revealed pronounced upregulation compared with controls (Fig. 7A–E). Silencing LRRK2 with siRNA reduced SASP expression to varying degrees (Fig. 7F). Treatment with IKK16 in the OE-LRRK2 and IL-1β + OE-LRRK2 groups reversed LRRK2-induced activation of the cGAS–STING–HMGB1 pathway, normalized senescence marker expression, and restored mitochondrial activity (Fig. 7G–J). These findings strongly indicate that LRRK2 is a key regulator of mitochondrial dysfunction and senescence in chondrocytes, acting through cGAS–STING–HMGB1 signaling.

Immunofluorescence co-localization further confirmed that LRRK2 and cGAS mutually enhance each other’s expression. cGAS expression was higher in the OE-LRRK2 group compared with controls (Fig. 5I–K). Furthermore, LRRK2 knockdown or IKK16 treatment significantly inhibited cGAS–STING–HMGB1 pathway activation and reduced TNF-α and MMP-3 expression (Fig. 7K–P). These results demonstrate that LRRK2 overexpression promotes mitochondrial dysfunction and accelerates chondrocyte senescence by activating the cGAS–STING–HMGB1 pathway, thus contributing to OA progression.

## Discussion

Chondrocyte senescence is increasingly recognized as a core pathological mechanism in OA, contributing to extracellular matrix degradation, metabolic impairment, and persistent SASP-mediated inflammatory signaling. Although multiple upstream stressors have been implicated in initiating this degenerative cascade, the molecular pathways linking mitochondrial dysfunction to senescence in chondrocytes remain poorly understood. In this study, LRRK2 was identified as a key regulatory molecule that bridges mitochondrial stress with senescence activation via a previously uncharacterized LRRK2–cGAS–STING–HMGB1 signaling axis. Our results show that LRRK2 functions not only as a stress-responsive kinase but also as an active promoter of senescence, therefore accelerating OA progression.

Transcriptomic profiling and mechanistic analyses showed that LRRK2 overexpression strongly activates canonical senescence pathways while simultaneously aggravating several key aspects of mitochondrial dysfunction, including organelle swelling, excessive ROS production, and impaired bioenergetic capacity. These mitochondrial disturbances were closely linked to senescence-associated mitochondrial dysfunction. Recent studies have also demonstrated that activation of the cGAS–STING pathway correlates with increased mitochondrial damage and enhanced cellular senescence [24]. Positioned as a mechanistic bridge, the cGAS–STING pathway connects LRRK2-induced mitochondrial instability to inflammation-driven senescence [18, 25]. Consistent with this, the classic senescence markers MMP-3 and BCL2 were significantly upregulated. These findings suggest that LRRK2 is not merely associated with general senescence processes but may actively initiate senescence by specifically engaging the cGAS–STING axis, linking LRRK2 signaling to cartilage degeneration and key drivers of chondrocyte senescence in OA.

Based on this model, the mechanistic cascade linking LRRK2 to OA-associated senescence was delineated. A previous study showing a relationship between Ras-related GTPase activity and HMGB1 expression [26] provides a strong basis for exploring whether LRRK2, through its GTPase domain, modulates this key DAMP to drive a senescent phenotype. HMGB1 is a multifunctional mediator involved in inflammation, senescence, and oncogenesis [27], and is also a major regulator of SASP production [28]. Thus, HMGB1 emerges as a pivotal downstream effector connecting LRRK2 signaling to the degenerative microenvironment of the OA joint.

The functional analyses identified the cGAS–STING pathway as the central intermediary in this mechanism. Activation of cGAS–STING led to a significant increase in HMGB1 expression and enhanced its nuclear-to-cytoplasmic translocation. Cytoplasmic HMGB1, in turn, contributed to mitochondrial dysfunction and amplified cellular senescence. The results show that LRRK2 regulates HMGB1 through both cGAS–STING activation and its intrinsic GTPase activity, revealing a dual mechanism of HMGB1 control that integrates mitochondrial stress responses with the execution of the senescence program.

Activation of the cGAS–STING pathway has been shown to play a pivotal role in cartilage degeneration in mouse models of joint disease [29]. Similarly, our in vivo results demonstrate that rAAV-mediated LRRK2 overexpression intensified chondrocyte senescence, accelerated cartilage matrix loss, and aggravated subchondral bone alterations in DMM-induced OA. Pharmacological inhibition of STING with H-151 partially reversed these effects, reducing HMGB1 accumulation, improving mitochondrial function, and mitigating cartilage damage. These demonstrate the LRRK2–cGAS–STING–HMGB1 signaling axis as a key molecular driver of senescence-associated cartilage deterioration.

An important strength of our study is the identification of HMGB1 as a key downstream effector of LRRK2 activation. HMGB1 release, characteristic of stressed or senescent chondrocytes, appears to play a central role in amplifying SASP signaling and reshaping the inflammatory microenvironment within the cartilage matrix. HMGB1 regulates oxidative stress, matrix-degrading enzyme expression, and persistent DNA damage responses, and its release is closely associated with elevated ROS production [30]. These functions position HMGB1 as a critical mediator that reinforces a self-perpetuating cycle of cellular senescence.

These results provide mechanistic insight into how mitochondrial dysfunction is converted into chondrocyte senescence and contributes to the establishment of a chronic, self-sustaining degenerative niche within the osteoarthritic joint. Because activation of the cGAS–STING pathway and HMGB1 upregulation are both closely linked to DNA damage, they may further potentiate senescence through DNA damage–dependent mechanisms [31, 32]. This observation highlights an important direction for future studies aimed at expanding our understanding of DNA damage–driven senescence in OA.

This study identifies a pathogenic LRRK2–cGAS–STING–HMGB1 signaling cascade that translates mitochondrial stress into a persistent senescent phenotype, accelerating cartilage degeneration. These findings broaden the recognized biological functions of LRRK2 beyond its established roles in neuroinflammation and position it as a key molecular amplifier of chondrocyte senescence in OA. From a translational standpoint, early disruption of this cascade, either by inhibiting LRRK2 or by blocking cGAS–STING signaling, may offer a promising senostatic strategy that slows OA progression while preserving tissue integrity.

STING inhibitors have already been explored across multiple inflammation-related disease models, including ischemia–reperfusion injury, pneumonia, and OA [33–35]. Thus, targeting LRRK2 upstream of the cGAS–STING–HMGB1 axis with inhibitors such as IKK16 represents a compelling therapeutic approach, and further investigation of this strategy may provide valuable insights for future OA research.

In future studies, it will be important to identify the upstream signals that elevate LRRK2 activity during joint degeneration, clarify the cell-type–specific roles of this pathway, and develop selective inhibitors that modulate LRRK2 activity in chondrocytes. These steps will be crucial for translating the mechanistic insights uncovered here into therapeutic advances. As senescence continues to gain recognition as a central driver of OA pathology, our findings position LRRK2 as a promising molecular target for reducing chronic inflammation, restoring mitochondrial stability, and slowing the progressive decline characteristic of OA.

Despite these advances, several limitations should be considered. First, LRRK2-deficient animal models were not employed to determine their essential role during the early phases of OA development. Second, although our findings show that LRRK2 overexpression disrupts mitochondrial function, the physiological triggers that elevate LRRK2 in human osteoarthritic cartilage remain unclear. Third, the long-term effects of pharmacological inhibition of LRRK2 on overall joint homeostasis are unknown, particularly given its established functions in other tissues.

## Conclusion

**Fig. 8:**
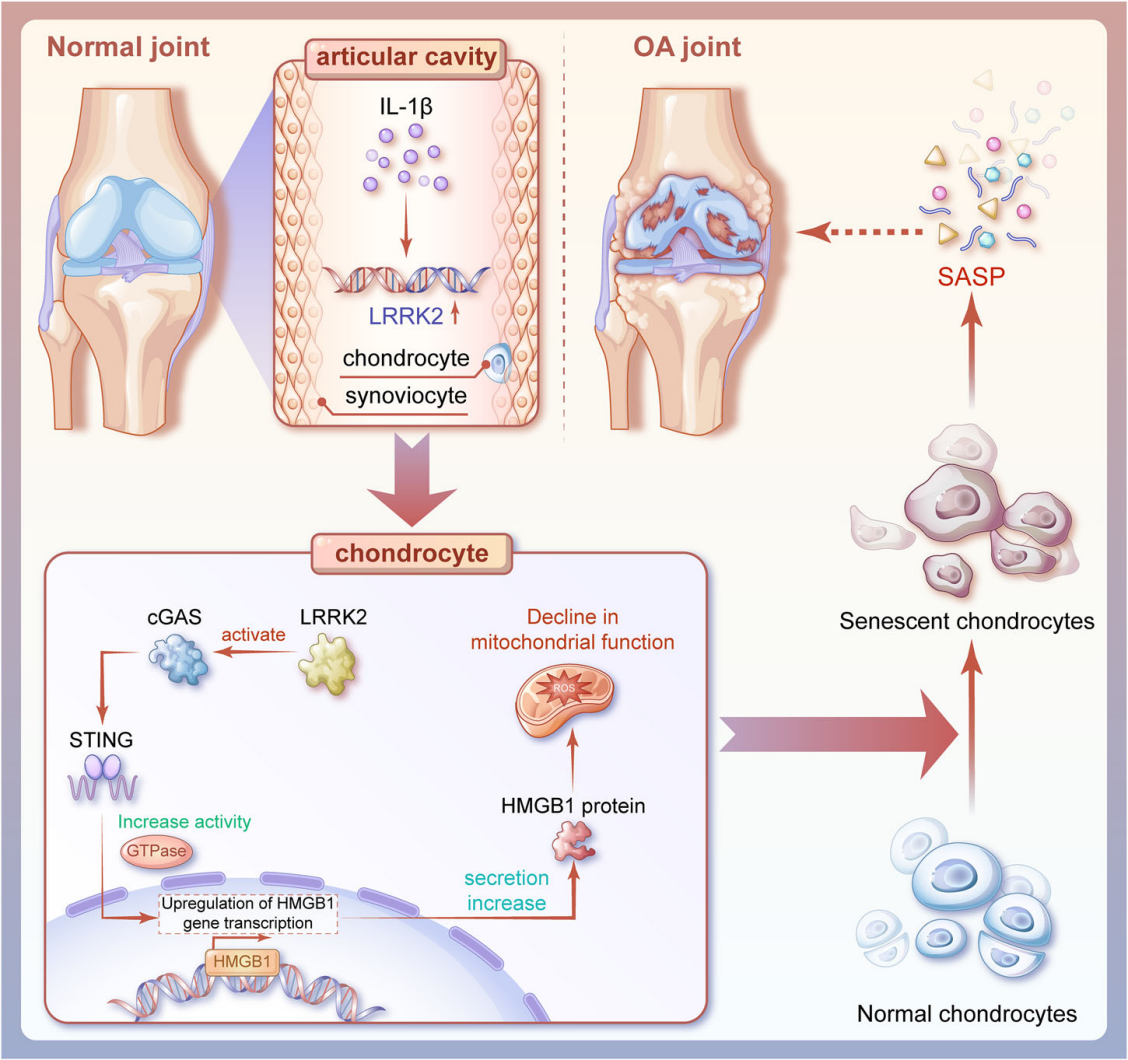
Hypothesis diagram of the mechanism by which overexpression of LRRK2 activates the cGAS–STING/HMGB1 pathway and promotes chondrocyte senescence, leading to OA.

This study clarifies the mechanism by which LRRK2 contributes to OA pathogenesis and confirms its pathogenic role. The results demonstrate that LRRK2 activates the cGAS–STING pathway, leading to increased HMGB1 production and subsequent induction of chondrocyte senescence (Fig. 8).

## Supplementary information

Supplementary WB figure

## Data Availability

The RNA sequencing and bioinformatics analysis datasets provided in this study are available at NCBI-Sequence Read Archive (https://www.ncbi.nlm.nih.gov/sra/). The accession code for deposited data is PRJNA1378519 and GSE286154.

## Code availability

The custom R scripts used for bioinformatics analysis, including differential expression analysis, GO/KEGG enrichment, and GSEA in this study, are publicly available on GitHub at [https://github.com/CebolaLab/RNA-seq]. Any additional analysis code is available from the corresponding author upon reasonable request.
